# Food policies for physical and mental health

**DOI:** 10.1186/1471-244X-14-132

**Published:** 2014-05-09

**Authors:** Felice N Jacka, Gary Sacks, Michael Berk, Steven Allender

**Affiliations:** 1 IMPACT Strategic Research Centre, Deakin University, School of Medicine and Barwon Health, PO Box 281, Geelong 3220, Australia; 2 WHO Collaborating Centre for Obesity Prevention, Deakin University, Geelong, Australia; 3 The University of Melbourne, Department of Psychiatry, Parkville 3010, Australia; 4 Orygen Youth Health Research Centre, 35 Poplar Rd, Parkville 3052, Australia; 5 The Florey Institute of Neuroscience and Mental Health, At Genetics Lane, Royal Parade, The University of Melbourne, Parkville 3010, Australia

## Abstract

Noncommunicable diseases (NCDs) account for the largest burden of early mortality and are predicted to cost the global community more than US $30 trillion over the next 20 years. Unhealthy dietary habits, in large part driven by substantial changes to global food systems, are recognised as major contributors to many of the common NCDs, including cardiovascular disease, cancer and diabetes. Recent evidence now indicates that unhealthy diets are also risk factors for mental disorders, particularly depression and dementia. This affords substantial scope to leverage on the established and developing approaches to the nutrition-related NCDs to address the large global burden of these mental disorders and reinforces the imperative for governments take substantial actions in regards to improving the food environment and consequent population health via policy initiatives.

## Background

According to the latest Burden of Disease report, noncommunicable diseases (NCDs), which include cancer, cardiovascular and circulatory diseases, chronic respiratory diseases, cirrhosis of the liver, digestive diseases, mental and behavioural disorders, neurological disorders, diabetes, urogenital, blood, and endocrine diseases, musculoskeletal disorders, and a multitude of other congenital and skin conditions, now account for more than half of Disability-adjusted life years (DALYs) globally [1]. DALYs are a measure of years of healthy life lost to death or non-fatal illness or impairment and represent a summary metric of population health [1].

Within the NCDs, mental disorders, including the ‘common mental disorders’ (CMDs) depression and anxiety, make up 7.5% of DALYs worldwide [1]. However, the substantial methodological difficulties in estimating the burden of mortality directly attributable to mental illnesses, as well as the paucity of available data [2] means that these figures are likely to under-represent the full picture of mental disorders burden. If we consider only disability, mental disorders account for 23% of health related disability worldwide, with unipolar depression accounting for the second highest number of years lost to disability. Similarly, dementias, which are also classified as mental disorders in the diagnostic rubrics, (although classified as neurological disorders in the Global Burden of Disease assessments) are imposing a major burden on societies, accounting for 11.3 million DALYs globally [1]. The incidence of dementia rises exponentially with age and there is expected to be a dramatic acceleration in the rates of people with Alzheimer’s disease or other forms of dementia worldwide as a function of the ageing population, with costs expected to escalate proportionately with numbers affected [3].

Many of these NCDs, including diabetes, cardiovascular disorders, and some cancers, are well known to be directly influenced by unhealthy diets; indeed, unhealthy diets are recognised as one of the major drivers of changes in the distribution and burden of NCDs over the second half of the 20th Century [4,5]. However, there is now nascent yet highly consistent evidence to suggest that unhealthy diet is also a key modifiable risk factor for some mental disorders, particularly CMDs and dementia. Importantly, in parallel to the obesity epidemic, there appears to be an increase in the prevalence of CMDs in a range of western countries, from the USA [6], Britain [7], Taiwan [8] and Australia [9]. We here argue that that there is an imperative to fully integrate mental disorders into the framework of NCD research and action and to make mental health an explicit target of policy designed to improve nutrition-related population health.

### Changes to the food supply and global impact on health

Substantial global changes in efficiencies of production, marketing, transport and sale of food (the food system) have had a highly detrimental impact on dietary patterns, with a global shift toward increased intake of fast foods and sugar sweetened beverages [10]. As a marker for the impact of food systems, obesity data provide a clear vantage point. The epidemic shows ongoing increases in the prevalence of obesity across adults in developed countries since the late 1970s, and more recent emergence of the epidemic in low and middle-income countries. Data from the UK [11] and detailed analysis of US Food data show that between 1960 and 1970 the available calories (expressed as energy available per capita per year) in the UK and US diet moved from a state of flux in the early 20th Century to a clear and relentless increase; importantly, these increasingly ’obesogenic’ environments explain much of the emergence of the obesity epidemic [5]. Equally, changes in economic conditions in low and middle-income countries, including increased economic wealth, the opening of trade markets to global partners and the shift from local production and distribution of food to transnational corporations, have led to rapid increases in diet-related NCDs. NCDs account for 63% of global deaths and are predicted to cost the global community more than US $30trillion over the next 20 years [12].

### NCDs and mental disorders

Somatic NCDs and mental disorders are highly comorbid and often mutually reinforcing, with many shared pathophysiological mechanisms and risk factors, including immune dysfunction (inflammation) and oxidative stress. Unhealthy diets, as well as physical inactivity and smoking, are established risk factors for systemic inflammation and oxidative stress, as well as for CMDs [13]. There is also emerging evidence regarding other important biological mediators of the diet-mental health association that are particularly pertinent to development and early life, including neurotrophic plasticity [14], disturbances in neurotransmitter systems [15], and epigenetic programming [16]. The most recent evidence supports an important role for gut microbiota, which are highly dependent on dietary intakes, in neurodevelopmental outcomes in animal models [17] and in depression in humans [18].

A parallel consequence of poor diet is obesity, which is a pro-inflammatory state. Obesity and depression share a well-established bidirectional relationship; obesity itself increases the risk for CMDs [19] and depression predisposes to the accumulation of excess adipose tissue [20]. Depression is associated with increased insulin resistance [21], as well as being an established risk factor for and consequence of cardiovascular disease [22,23]. Similarly, type 2 diabetes [24], hyperinsulinemia [25], high BMI [26], and hypercholesterolemia [27] are all risk factors for dementia and cognitive decline and clearly influenced by dietary habits. Even raised blood glucose at the high end of the normal range is now established as a risk factor for dementia [28]. Thus, many of the same pathways whereby poor dietary practices contribute to the high prevalence of NCDs influence the risk for and progression of mental disorders [29].

### Diet and mental health

Since the end of 2009, there has been an exponential rise in the number of published studies examining the possible influence of habitual diet on the risk for CMDs. Both cross-sectional and prospective studies documenting associations between higher diet quality and a reduced likelihood or risk for CMDs, as well as an increased likelihood or risk for CMDs with higher intakes of unhealthy food products, have been published from countries across the globe in children, adolescents and adults [30]. A recent meta-analysis has confirmed that adherence to a ‘healthy’ diet pattern is associated with a reduced likelihood of depression in adults. Although studies were too few for confirmation, there was a strong trend also observed for a positive relationship between ‘western’ style (unhealthy) dietary pattern and depression [31]. Similarly, another recent meta-analysis reported that high adherence to a Mediterranean diet, also a very healthful dietary pattern, is associated with a 30% reduced risk for depression [32].

Poor diet quality is also clearly associated with mental health problems in young people, independently of important familial factors and other health behaviours (eg. [33,34]). Of particular note are the most recent data from a very large Norwegian cohort study, indicating that maternal diet during pregnancy, as well as dietary patterns during the first years of life, are associated with an increased risk for mental health problems in very young children [35]. In this study, there was evidence suggesting consistently great effects of unhealthy diets on children’s mental health outcomes compared to the effects of insufficient healthy food intake. A recent systematic review has now confirmed a relationship between ‘unhealthy’ dietary patterns and poorer mental health in children and adolescents [36]. Given that CMDs tend to first manifest in childhood and early adolescence [37], these data suggest that unhealthy diets in pregnant mothers, children and adolescents, may be an important modifiable exposure increasing the risk for mental health problems across the lifespan.

Similar findings are also seen in studies examining the impact of dietary behaviours on dementia and cognitive decline in older adults. As noted previously, nutrition-related health conditions are risk factors for dementia and cognitive decline. High saturated fat intake appears to increase the risk for cognitive decline, while higher intakes of monounsaturated fats seem to be protective [38]. Higher adherence to a Mediterranean dietary pattern, as well as other forms of healthful diet, is associated with a reduced risk for Alzheimer’s disease and cognitive decline [39-42]. Indeed, a recent meta-analysis reported a 40% reduction in the risk for incident cognitive impairment for those with high adherence to a Mediterranean diet [32]. While the most recent review of the topic, commissioned by Alzheimer’s Disease International, concluded that there is little empirical evidence to support nutritional supplementation to prevent or treat cognitive problems, they noted a strong theoretical basis for implicating micronutrient deficiencies in the known mechanisms of neurodegeneration [3].

Although the evidence base for the relationship between dietary intake and mental disorders is very new, and thus largely limited to animal studies and observational studies in humans to date, a recent large-scale dietary intervention provides important support for the findings of the meta-analyses. In the PREDIMED study [43], individuals at increased risk for cardiovascular events were randomised to a Mediterranean diet supplemented with either extra-virgin olive oil (EVOO) or mixed nuts, with a low-fat control diet. Although not statistically powered to assess prevention of depression, the results demonstrated a strong trend to a reduced risk for incident depression for those randomised to a Mediterranean diet with nuts, and this protective effect was particularly evident in those with type 2 diabetes [44]. Those in the Mediterranean diet groups also demonstrated improved cognition compared to controls [45]. This is the first study to provide support for dietary intervention as a strategy to prevent mental disorders. Randomised controlled trials to test causal relationships between dietary improvement and improvements in mental health are required and currently underway [46].

Taken together, these data indicate that the changes in dietary habits globally are likely to be influencing the prevalence of CMDs and dementia. Moreover, the impact of these dietary patterns on the global burden of CMDS and dementia may not yet be fully manifest, given that the detrimental changes to food intakes are particularly obvious in younger generations [10,47]. The implications of detrimental changes in the food landscape may thus be formidable.

### Food policy for physical health

In recognition of the substantial burden of lifestyle-related NCDs, the World Health Organization (WHO) developed the Global Strategy on Diet, Physical Activity and Health (DPAS) [48] in 2004. DPAS identified the need for integrated multi-sectoral policy action to improve food environments and population diets, and highlighted the need for government policy intervention at regional, national, and local levels. The need for policy action to address the growing NCD burden gained further prominence in 2011 when the United Nations (UN) General Assembly adopted the *Political Declaration of the High-level Meeting of the General Assembly on the Prevention and Control of Non-communicable Diseases* (UN Political Declaration on NCDs) [49]. The UN Political Declaration on NCDs acknowledges the direct impact of NCDs on social and economic development and provides a strong impetus for governments take preventative actions and demonstrate sound leadership. As with DPAS, the UN Political Declaration on NCDs recognises that effective NCD prevention requires the involvement of many different stakeholder groups, and multi-sectoral approaches involving, amongst others, health, education, agriculture, industry and trade, and finance [49]. WHO has subsequently developed a set of nine voluntary global NCD targets, including a target of a 25% reduction in premature mortality from NCDs by 2025 [50]. However, none of the other eight targets and 25 indicators set by WHO explicitly include mental health. Moreover, mental disorders are not included as measured outcomes when assessing the impact of policy changes on population health parameters.

Specific food policy recommendations to address NCDs typically include two components: (1) embedded structures within government to support food policy and NCD prevention interventions; and (2) population-wide policy initiatives that help to create environments that support healthy diets. The first component includes leadership, ‘health-in-all’ policies, dedicated funding for population nutrition promotion, monitoring systems, workforce capacity, and partnerships that need to be in place in order to support and enhance the effectiveness of the more direct policy initiatives [51]. The second component includes laws, regulations, taxes and subsidies that affect various aspects of food systems [52]. Policies likely to be effective interventions include restrictions on the marketing of unhealthy foods and beverages to children, improved and interpretive nutrition labelling (e.g. using colours or stars on the front-of-pack), and taxes on unhealthy foods (e.g. sugar sweetened drinks) [53]. While it is clear that these policy actions are not currently designed specifically to address mental health problems, they may serve as ‘stealth interventions’ [54] to address CMDs indirectly. However, we would argue that more direct action is now needed to directly address mental health via nutrition-related policy and initiatives.

### Food policy for mental health

While the informal definition of NCDs ‘usually excludes mental illnesses’ [55], there is now clear and consistent evidence to suggest that segregation between high prevalence mental disorders and nutrition-related NCDs may be artificial and erroneous. As such, we would argue that it is now time to explicitly integrate CMDs and dementia into the broader category of lifestyle-related NCDs. Where initiatives targeting cardiovascular disorders, obesity and diabetes prevention gain traction, the possible benefits for mental disorders need to be identified and quantified. Furthermore, food policy researchers should consider incorporating mental health outcomes into their evaluations. For example, while previous cost-effectiveness evaluations of population-wide interventions that successfully improve dietary behaviours have not incorporated mental health outcomes [53], it is likely that the economic credentials of the interventions would improve if mental health outcomes were also taken into account. It is also critical that CMDs and dementia are included as measured outcomes when initiatives and policy changes designed to improve population health are implemented and evaluated.

Preventive and public health messages and educational programs need to be developed in partnership with a range of public health, clinical and advocacy groups to ensure that the general public, clinicians and policy makers are aware of the links between dietary behaviours and mental health. An explicit recognition by the public of the importance of dietary risk factors to the CMDs and dementia would be of substantial value in reinforcing policies at a government level. Similarly, although not directly a policy issue, ensuring that information, education and support for dietary improvement is offered as part of integrated treatment packages for those with mental health problems would seem to be an important component of this equation. Although quality evidence on dietary improvement as a treatment strategy for CMDs is not yet available, the strength of the evidence from observational studies and the nascent data from preventive interventions would offer a strong basis on which to make explicit recommendations for clinical practice via official guidelines. Such recommendations are in line with those for somatic illness and will, at the very least, address the common physical comorbidities in mental disorders. It is also possible that messages regarding mental health may have more valence to and influence on the health behaviours of patients and the general public, given that mental health symptoms are often a more proximal than the more distal risk of future health conditions such as heart disease, cancer and diabetes. Finally, when mental health advocates develop interventions and recommendations for prevention, policy and practice, nutrition-related health promotion messages, as well as support for appropriate taxation, labelling, monitoring and other nutrition-related policy initiatives, should be included.

## Conclusion

Given the limited traction gained in the fight against the unhealthy commodities industries to date, it would be unrealistic to expect substantial gains in the short term. However, because of the scale of the burden of illness of CMDs and dementia and the universality of food as a modifiable risk factor, the benefits of even small improvements in the food environment, leading to improved dietary intakes, may translate to large gains in mental health at a population level. As such, there is an imperative to integrate mental health into the framework of NCD research and action in order to leverage on the established and developing approaches to the NCDs and explicitly evaluate mental health outcomes that may arise as a function of such initiatives.

## Competing interests

The authors declare that they have no competing interests.

## Authors’ contributions

FJ conceived of the paper and participated in its design and coordination and helped to draft the manuscript. GS, MB and SA provided important intellectual content and participated closely in the drafting of the manuscript.

## Pre-publication history

The pre-publication history for this paper can be accessed here:

http://www.biomedcentral.com/1471-244X/14/132/prepub
